# On the Crucial Cerebellar Wound Healing-Related Pathways and Their Cross-Talks after Traumatic Brain Injury in *Danio rerio*

**DOI:** 10.1371/journal.pone.0097902

**Published:** 2014-06-13

**Authors:** Chia-Chou Wu, Tsung-Han Tsai, Chieh Chang, Tian-Thai Lee, Che Lin, Irene Han-Juo Cheng, Mu-Chien Sun, Yung-Jen Chuang, Bor-Sen Chen

**Affiliations:** 1 Deptartment of Electrical Engineering, National Tsing Hua University, Hsinchu, Taiwan; 2 Deptartment of Medical Science and Institute of Bioinformatics and Structural Biology, National Tsing Hua University, Hsinchu, Taiwan; 3 Institute of Brain Science, National Yang-Ming University, Taipei, Taiwan; 4 Stroke Center and Deptartment of Neurology, Changhua Christian Hospital, Changhua, Taiwan; MGH, MMS, United States of America

## Abstract

Upon injury, the direct damage and the subsequent secondary injury in the brain often result in chronic neurological disorders. Due to multifactorial nature of secondary injury and subsequent complex cellular responses, much of the underlying mechanisms are unclear. This study used an adult zebrafish cerebellum injury model to investigate the phenotypes and the secondary injury responses for recovery mechanisms of injured brain. Using the time course microarray analysis, a candidate protein-protein interaction (PPI) network was refined as cerebellar wound healing PPI network by dynamic modeling and big data mining. Pathway enrichment and ontological analysis were incorporated into the refined network to highlight the main molecular scheme of cerebellar wound healing. Several significant pathways, including chemokine, Phosphatidylinositide 3-kinases, and axon guidance signaling pathway and their cross-talks through PI3K, PAK2, and PLXNA3 were identified to coordinate for neurogenesis and angiogenesis, which are essential for the restoration of the injured brain. Our finding provides an insight into the molecular restoration mechanisms after traumatic brain injury, and open up new opportunity to devise the treatment for traumatic brain injury in human.

## Introduction

Traumatic brain injury (TBI), also known as intracranial injury, is a major cause of death and disability worldwide, especially in children and young adults. Recent data have shown that approximately 1.7 million people sustain a TBI annually in the United States [1]. TBI is caused by an impact on the head or a penetrating head injury that disrupts normal brain function. Upon injury, the direct damage and the subsequent secondary injury in the brain often results in chronic neurological disorders. The multifactorial nature of secondary injury after brain injury is characterized by a complex multi-cellular process that involves apoptosis, inflammation, proliferation of glial cells and increased progenitor cell activity, which can lead to an increase in neurogenesis [2]. However, much of the underlying molecular restoration mechanisms are still unclear and this leads less effectively therapeutic strategies for TBI in human.

The central nervous system (CNS) is crucial to the most organisms. Once damaged, there would be lethal effects if it cannot be healed or regenerated. Recently, advances in neuroscience research have led to the development of innovative therapeutic strategies that aim to regenerate damaged CNS. Tissue regeneration is one of the most interesting biological phenomena. However, the molecular and cellular mechanisms by which regeneration takes place are still unclear [3]. The ability to regenerate lost or damaged parts of the central nervous system (CNS) is seen in varying degrees in many organisms. In the past, the adult mammalian CNS was viewed as a system without the capacity for regeneration once damaged [4]. In contrast to mammals with their limited ability to regenerate CNS as adults, *Danio rerio* (zebrafish) can constitutively produce new neurons along the rostrocaudal brain axis throughout its lifespan. Other organs also have an extensive regenerative capacity and respond to injuries such as trauma, lesion and ischemic episodes by producing new tissues to replace the lost ones. Adult zebrafishs have the extraordinary ability to regenerate their damaged fins, skin and heart [5], [6]. Furthermore, they can regenerate several organs or tissues in the nervous system: spinal cord, photoreceptor, retina, cerebellum and optic nerve [6]–[11]. This feature of the adult zebrafish brain relies on the presence of neural stem cell niches that enable stem cells to continuously proliferate, and on a permissive environment for neurogenesis in the brain [12]. Additionally, the zebrafish immune system is also remarkably similar to mammalian immune systems. Overall, the zebrafish genetic map demonstrates a highly conserved synteny, similar to the human genome [13]. To this end, the zebrafish has emerged as a powerful vertebrate model for elucidating the molecular and cellular mechanisms of regeneration [5], and numerous studies have already used this species to study the regeneration of the nervous system [12].

In the present study, the schematic diagram of TBI experiment on zebrafish by stab lesion is established and the gene expression levels are measured through time course microarray experiments from injury to recovery stage. With the time-course microarray experiments, the molecular mechanisms of the cerebellar wound healing are studied using the dynamic network modeling [14]. The dynamic network model integrates the information from various protein-protein interaction (PPI) databases and the time-course microarray data in this study. The resultant PPI network serves as a basis to illustrate the molecular mechanisms of cerebellar wound healing. Meanwhile, the evolution of behavior of zebrafish from injury to recovery is observed under the confocal microscope, video-recorded, and quantitatively analyzed to measure the degree of disability caused by the injuries. This analysis would be helpful to focus on those genes involving the recovery process if a high correlation is shown between the gene expression profile and the ZMI. According to the gene expression profiles and the ZMI, we focus on three groups of genes, i.e., the acutely activated, positively correlated, and negatively correlated to ZMI groups. To extract knowledge from the time-course expression data, several systems biology tools, e.g. STEM [15] and PANTHER [16], were adapted in this study besides the dynamic network modeling. Several significantly enriched pathways in the above three groups are identified, e.g. chemokine signaling pathway (inflammation-related), Phosphatidylinositide 3-kinases (PI3K) signaling pathway (cell cycle-related), and axon guidance pathway. Then, using the dynamic cellular PPI network as a backbone, the cross-talks to coordinate with these pathways during the recovery process and the schematic diagram of wound healing-related cellular pathways are also presented in this study. Plausible nodes within the network of signaling pathways of the injured cerebellum were PI3K, PAK2, and PLXNA3 for coordination of neurogenesis and angiogenesis, which are essential for the restoration of injured brain. Moreover, we found these pathways may stimulate sub-networks important for neurogenesis and angiogenesis.

These findings not only confirm that our methods were successful, but also improve the confidence on the wet experiment design employed. Some interactions, such as cross-talks to coordinate among pathways for wound healing, were identified in this study, which will aid in deciphering these complex molecular restoration mechanisms in the future. Our finding provides an enhanced understanding of the molecular restoration mechanisms after a traumatic event to the brain, and open up new opportunity to devise treatment for traumatic brain injury in human.

## Materials and Methods

### Stab lesion assay and time course microarray experiments in zebrafish TBI model

**Figure 1:**
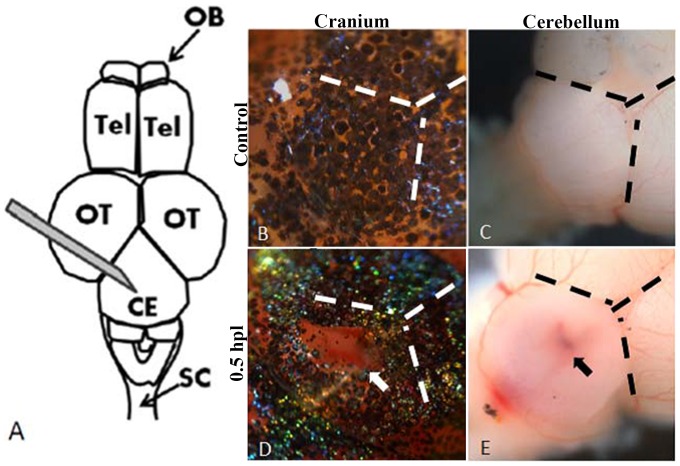
The diagram of the stab lesion assay. A: Schematic diagram of the stab lesion. A 27G syringe was used to create a cranial wound of depth 1.5 mm in the cerebellum (OB, olfactory bulb; Tel, telencephalon; OT, optic tectum; Ce, cerebellum; SC, spinal cord). B–E: Bright field images of the cranium (B, D) and exposed brains (C, E) of control fish and after the stab lesion. B and C show intact cranium and cerebellum of the control (no stab lesion) and D and E show the injured cranium and cerebellum of an experimental animal at 0.5 hour post-lesion (hpl). The fresh wound can be seen clearly on both the cranium and the cerebellum (D, E; the white arrow shows the lesion site on the cranium and the black arrow shows the wound on the exposed cerebellum).

Zebrafish line (Tg(kdr:EGFP)) and wildtype were used to perform the stab lesion assay, microarray, and behavior video tracking experiments. The body length of all zebrafishes used in this study were 2.8–3.8 cm. Before the stab lesion assay, the zebrafishes were kept under a light cycle of 14 h light and 10 h dark at a temperature of 28°C. In the stab lesion assay, six-month-old adult zebrafish were anesthetized by immersion in the aquarium water in which 200 ppm tricaine (MS222; Sigma, St. Louis, MO, USA) is dissolved for 5 minutes. A syringe (27G; Thermo Scientific, USA) was punched vertically through the cranial surface into the zebrafish cerebellum to a depth of 1.5 mm (Fig. 1). There are 3 large scales on the skull, which perfectly match with the positions of 2 optic tectum hemispheres and the cerebellum. The accumulating pigment around the 3 scales could also help us locate the lesion site of the wild-type zebrafish's brain. The injury depth is controlled by wrapping the needle with plastic tube and only left 1.5 mm needle tip for stabbing. Also the lesion depth with sagittal brain sections is examined (see Fig. S1 in File S1). The injured zebrafish were then put back into fresh aquarium water for recovery. Zebrafish that had apparently ceased bleeding were selected for the subsequent experiments. The control group is not anesthetized. Every fish in the experiment is only anesthetized with tricaine to cause the lesion except the control animals. The tricaine anesthetization is the standard procedure in zebrafish experiments. After lesion, the whole sample preparation process does not use any anesthetic drugs. Before they were sacrificed, we used ice-cold water to immobilize them and quickly dissected the cerebellum out. The dissected cerebellum was collected into a 1.5 ml microcentrifuge tube with 200 µl TRIzol (Invitrogen, Carlsbad, CA, USA) (8 cerebellums per tube). After homogenization, we filled each tube with TRIzol up to 1 ml. After lesion, the whole sample preparation process does not use any anesthetic drugs. Therefore, the anesthetic process will not cause much influence on the gene expression profiles. The experimental procedures were approved by the committee for the use of laboratory animals at National Tsing-Hua University (IACUC number: 10140).

**Figure 2:**
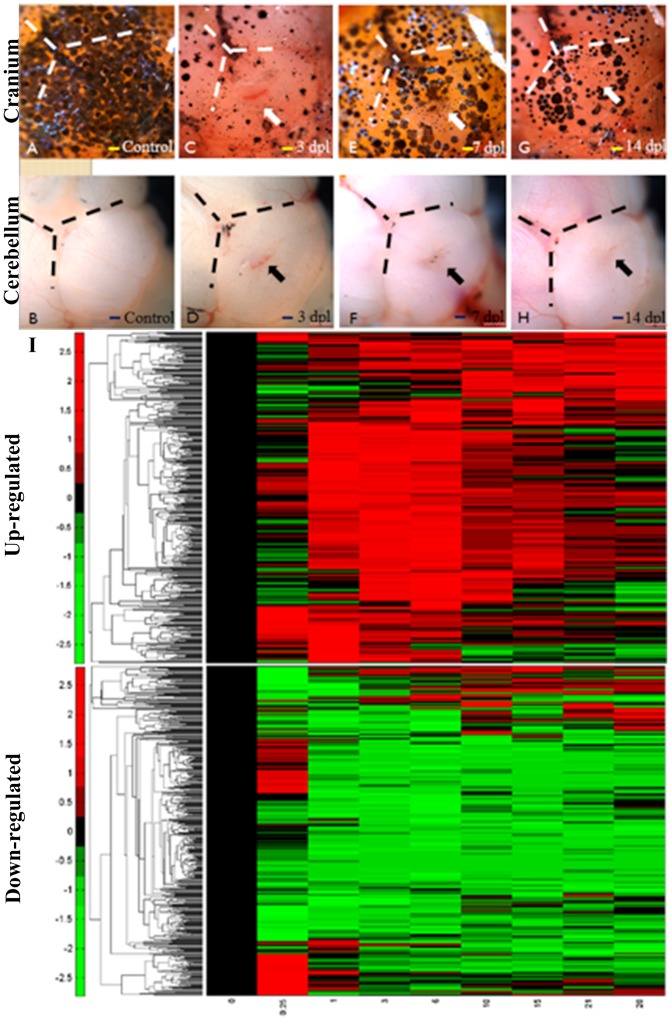
Regeneration of cerebellum at different day-post-lesion and the time-course microarray data in cerebellar wound healing process. A-H: Bright field images of the craniums (A, C, E, G) and cerebellums (B, D, F, H) at different time points post lesion. The cranial and cerebellum were intact before stab lesion (A, B). At 3dpl, the wound can be seen on the cranial and cerebellum. (C, D) At 7dpl, the cranial is seal and scar is observed in the cerebellum. (E, F) At 14 dpl, the scar was hardly seen. (Scale bar, 100 µm.). (I) The differentially expressed genes (≥1.5 or ≤0.67 fold change) are hierarchically clustered (5839 genes). Columns represent the day-post-lesion (dpl), and the rows represent the genes. The blocks indicate temporally up-regulated and down-regulated genes, respectively. The color bar represents the log value of the ratio relative to the intensity at 0 dpl.

For the microarray experiments, zebrafish were euthanized by prolonged immersion in ice-cold water. Then, the zebrafish were dissected quickly to harvest the brains. Each dissection took approximately 4 minutes. A surgical knife was used to cut out the cerebellums. The separated cerebellums were kept in RNAlater (Ambion, Inc., Austin, TX, USA). We collected the cerebellums at the following time points: Control (no injury), 0.25, 1, 3, 6, 10, 15, 21 and 28 days post-lesion (dpl) (The recovery process is also monitored using immunohistochemistry staining; see Fig. S2 in File S1.). At each time point, the sample consisting of eight cerebellums from male zebrafishes. Microarray analysis of the samples was conducted by WELGENE Biotech CO., LTD. The quality assurance and quality control data for the samples are shown in Table S1 (in File S1). The regeneration of cerebellum and the time course microarray data in the cerebellar wound healing process are shown in Fig. 2. The microarray dataset discussed and used to reconstruct the PPI network in this study can be retrieved as Dataset GSE56375 from the NCBI GEO repository website (http://www.ncbi.nlm.nih.gov/geo/).

### Zebrafish Movement Index (ZMI) experiments

**Figure 3:**
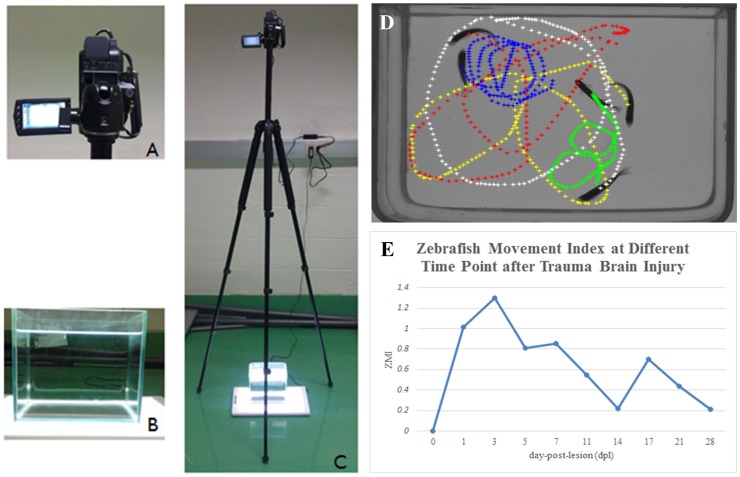
Equipment setup for behavior analysis. A: Close-up views of the video camera. B: Close-up views of the aquarium. C: The camera and aquarium setup. D: Swim sample paths of zebrafish after TBI. E: Quantitative behavior index (zebrafish movement index, ZMI) of injured zebrafish describes the degree of disability in the behavior of the injured zebrafish. The greater the index value, the greater the degree of disability.



For the ZMI experiments, zebrafish were placed in a glass tank (18×12×15 cm) filled with 2.5 L aquarium water. For each time point, 3 male adult zebrafish (with body length ranging from 2.8 to 3.8 cm) were transferred to the observation tank. Behavior was recorded with a video camera (HDR-XR200; Sony, Japan) which was placed above the tank and set to record trails of 5 minutes duration (Fig. 3A–C). The first video of zebrafish behavior was recorded after placing the zebrafish in aquarium water for 25 minutes, and the second video of zebrafish behavior was recorded after a 10 minutes break. We have chosen three behavioral patterns as criteria for evaluating the movement of zebrafish, including total swimming distance (distance index, DI), single direction turning (turn direction index, TDI), and turning angle (turn angle index, TAI) [17], [18]. The formulae of each index are in the following with the notations: C and I stand for control and injured fish, respectively.

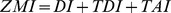
where
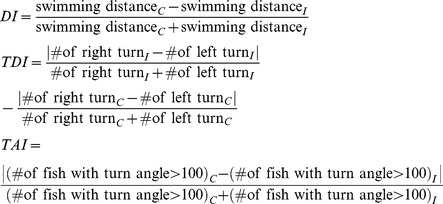



Note that the values of the three indices are between 0 and 1 and would be equal to 0 when the behaviors of injured fish perfectly match to control fish. We used Multiple Zebrafish Tracking and Behavior Analysis software from Dr. YC Chen's Laboratory (Department of Electrical Engineering, NTHU) to analyze the swimming distance, turn direction, and turn angle for each zebrafish in the video clips. An example of a swim path diagram used for this behavior analysis is shown in Fig. 3D.

### Big data mining for candidate PPI network

**Figure 4:**
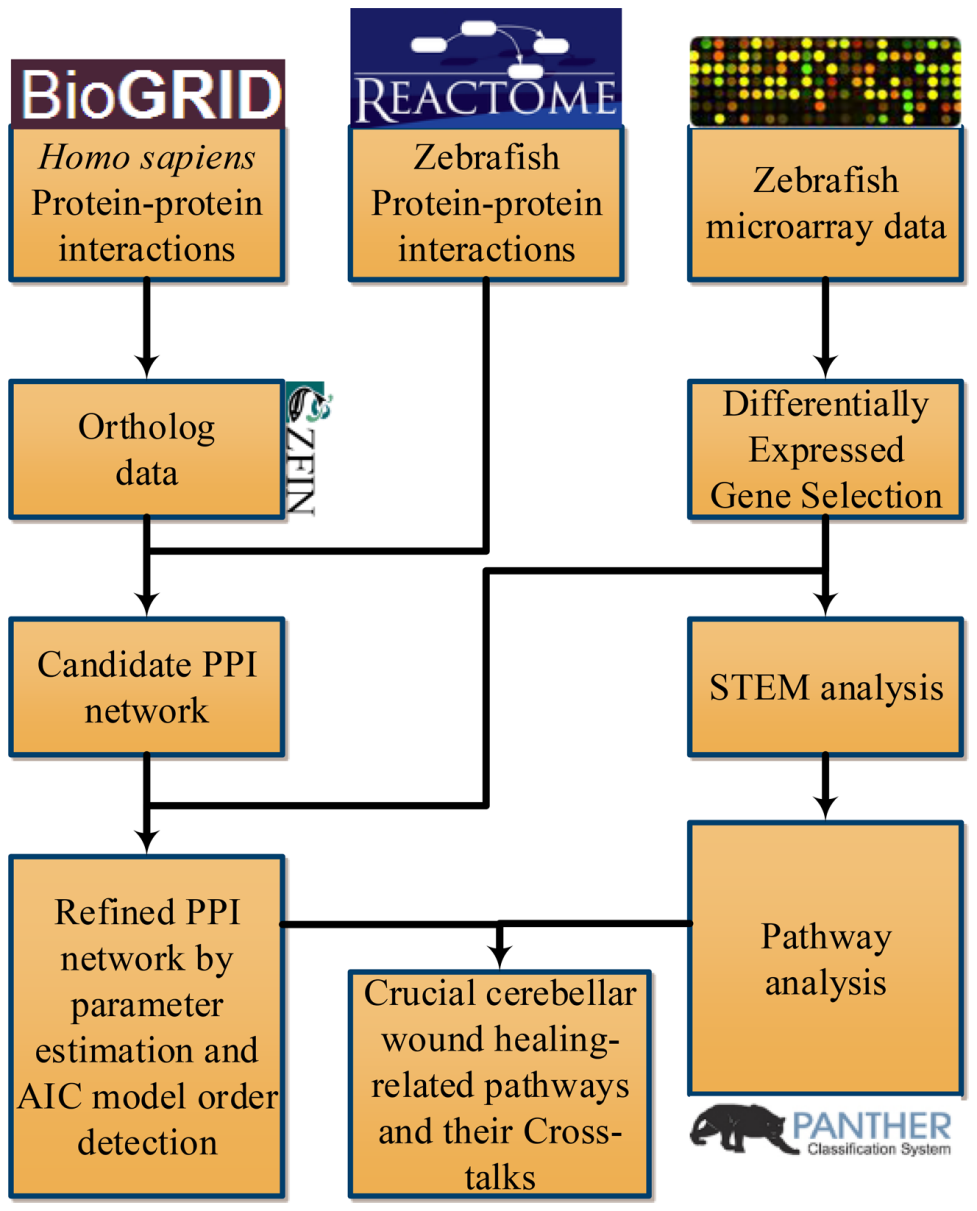
Flowchart for constructing the wound healing-related PPI network using a dynamic network model and big data mining. The network is constructed by PPI information from databases mining and systems identification according to the time-course microarray data (as shown in the boxes). Open access systems biology tools STEM and PANTHER are used to identify significantly temporal patterns and enriched cerebellar wound healing-related pathways.

Several types of data are integrated to construct the cerebellar wound healing molecular network, including time course expression profiles (Fig. 4), zebrafish and human PPIs (from BioGRID and Reactome) [19], [20], zebrafish-human gene ortholog data (ZFIN) [21], and zebrafish gene annotations were retrieved from Gene Ontology (GO) [22]. The PPIs of human and zebrafish collected from the databases are served as a collection of candidates of potential PPIs in the cerebellar wound healing network. However, there is a paucity of information on zebrafish PPIs. Hence, we inferred part of zebrafish PPIs from human PPIs based on ortholog information. Then, we had PPI information (inferred from human+zebrafish) and time course expression profiles. For setting up the protein pool for differentially expressed proteins, these expression profiles were used to select differentially expressed proteins according to fold change. The differentially expressed proteins were identified if their fold changes were ≥1.5 or ≤0.67 comparing to control time point. These differentially expressed proteins were assumed to be most likely involved in the cerebellar wound healing process. Based on the protein pool and PPI information, a candidate PPI network for the cerebellar wound healing process was constructed.

### Dynamic network modeling for cerebellar wound healing PPI network

**Figure 5:**
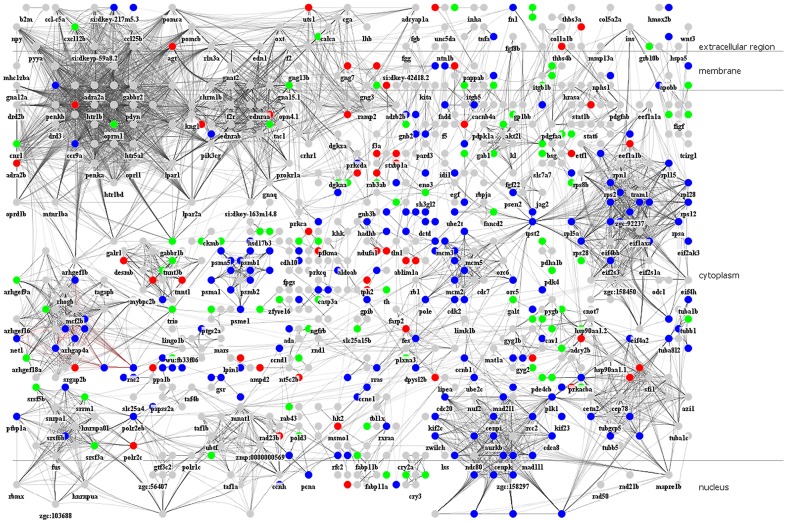
The constructed zebrafish cerebellar wound healing-related cellular PPI network by dynamic network modeling via microarray data and big data mining. The cerebellar wound healing-related PPI network of zebrafish contains 5270 PPIs among 802 proteins. The red, green, and blue nodes belong to group N, A, and P (see Fig.6 and the context for details). The information to draw the network is summarized in the File S2.

However, since the candidate PPI network was based on a wide variety of biological experimental conditions and/or ortholog information, it may have contained many interactions irrelevant to the cerebellar wound healing process (i.e. false positive interactions). To develop a realistic PPI network for the cerebellar wound healing process, the candidate PPI network was needed to further prune and then obtain a refined PPI network (see Fig. 5 and the File S2 for detailed information) by system identification method and system order detection method using the real microarray data, and the dynamic network model in the following:
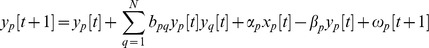
where *y_p_*[*t*] and *y_q_*[*t*] represent the protein activity level of the target protein *p* and the *q*th protein interacting with *p* at time *t*, respectively; *b_pq_* denotes the ability of the *q*th interactive protein to interact with *p*; *α_p_* denotes the translation effect from mRNA to *p*; *x_p_*[*t*] represents the mRNA expression level of *p*; *β_p_* indicates the degradation effect of *p*; and *ω_p_*[*t*+1] is stochastic noise. Rewriting the above dynamic equation with vector notation, we get:
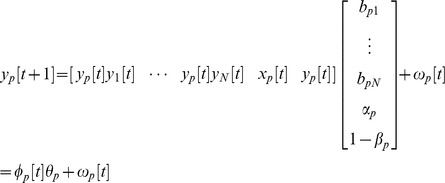



By collecting all the data from every time point, the interactions between the target protein *y_p_* and its candidates (*y*_1_,⋯,*y_N_*) can determined through the system identification and system order detection methods. The technique details are described in methods S1, S2, and S3 in File S1. To this end, the final PPI network are completed.

### Systems biology tools and statistics

**Figure 6:**
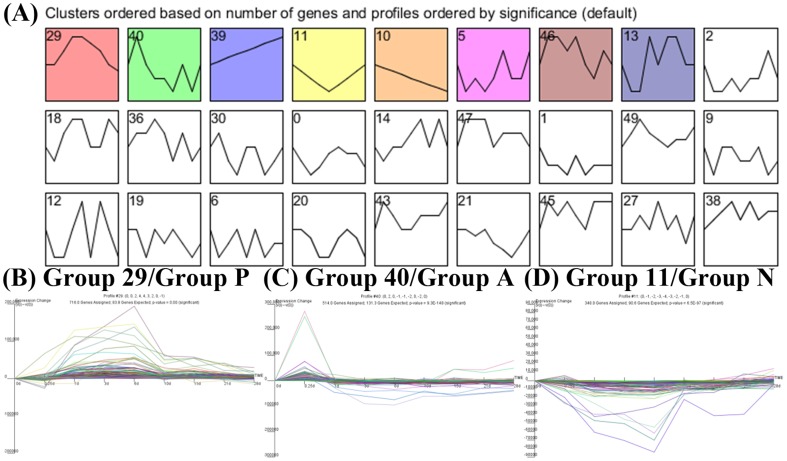
Significantly temporal patterns in the wound healing process. (A) Using STEM, significantly temporal patterns are identified (color background) (FDR corrected p-value <0.05). According to the STEM results and ZMI, we focused on group 29, 40, and 11, which are positively correlated with ZMI (B), acute response (C), and negatively correlated with ZMI (D).

After refining the candidate PPI network, two open access programs were used to analyze the time course expression profile. For identifying the significant patterns of time course expression profiles, The STEM tool is used to identify significant temporal patterns from time-course expression levels data. The default settings (the default enrichment in STEM is actual size based enrichment, in which the enrichment is computed using the hypergeometric distribution based on the number of genes in the set of interest) are used for the analysis and the significant patterns (with the Bonferonni corrected p-value <0.05) in Fig. 6A are highlighted with color background and ordered by the corrected p-value.

For identifying significantly enriched functions in the specific pattern groups identified by STEM, we performed enrichment and ontology analysis through the website of Protein Analysis Through Evolutionary Relationships (PANTHER) [16]. This tool can map gene lists to GO molecular function and biological process categories, as well as PANTHER biological pathways. Furthermore, it can display the results in pathway diagrams to enable visualization of the relationships between genes in known pathways. The PANTHER tool is used to identify the significantly enriched pathways in a group of proteins or genes. The default settings (the binomial test is applied to determine whether there is a statistical overrepresentation or underrepresentation of the genes and/or proteins in the test list relative to the reference list provided by PANTHER) are used for the analysis and the Bonferroni correction for multiple testing is used for correcting the original p-value. The significantly enriched pathways are with the corrected p-value<0.05.

The sign test is applied to justify the strong correlation between the temporal expression profiles and ZMI at the 5% significance level, where the null hypothesis is the probability of having positive correlation is equal to 0 (i.e., the median of the Pearson correlation coefficients between the temporal expression profiles and ZMI is zero) and the alternative hypothesis is the probability of having positive correlation is not equal to 0 (i.e., the median of the Pearson correlation coefficients between the temporal expression profiles and ZMI is not equal to zero).

## Results

### The significantly temporal patterns and PPI network for the cerebellar wound healing process in the zebrafish TBI model

The first principal aim of this study was to construct an integrated cerebellar wound healing-related cellular PPI network. Various types of data are integrated with dynamic network model and microarray data under this framework (Fig. 4). There are 802 proteins (nodes) and 5270 interactions (edges) in the final PPI network (i.e., refined PPI network by AIC system order detection; Fig. 5). This PPI network serves as the backbone of our further analysis. Information from different systems biology tools can be integrated under this framework. First, the temporal information from the time course microarray data in Fig. 2 are used to build up the PPI network through dynamic network modeling. The time course microarray data is compared to 2 published data using zebrafish as a model system to study the regeneration of neural systems (spinal cord and optic nerve) [10], [23]. We found our data is highly consistent with the reported findings (the correlation coefficients are about 0.5∼0.7) although the experimental conditions (the lesion schemes) and the studying objects (the injured parts of neural systems) are different (see Fig. S3 in File S1). Thus, this can serve as an independent validation of our results. Next, we use STEM to find significantly temporal patterns in time course microarray data. Then, several significantly temporal patterns are revealed (Fig. 6). The temporal pattern of group 29 (group P hereafter) showed a strong *p*ositive correlation with the ZMI (comparing Fig. 3E with Fig. 6B, the Pearson correlation coefficient is 0.7427±0.1315 with the p-value for sign test is less than 0.05). The temporal pattern of group 11 (group N hereafter) was the reverse, i.e. there was a highly *n*egative correlation with the ZMI (comparing Fig. 3E with Fig. 6D, the Pearson correlation coefficient is −0.7050±0.1167 with the p-value for sign test is less than 0.05). The temporal pattern of group 40 (group A hereafter) was related to *a*cute responses during the wound healing process (Fig. 6C) since the variations of expression levels are aroused earlier than group P and N [24], [25].

Second, qualitative functional information were integrated to investigating the molecular systems mechanisms of cerebellar wound healing process. For the genes in group A, some of the most common gene ontology (GO) biological processes were related to the regulation of MAP kinase activity, regulation of the MAPK cascade, phosphorylation, and dephosphorylation. For the genes in group P, some of the most common GO biological processes were related to the regulation of the cell cycle, meiosis, DNA replication, and organelle organization. For the genes in group N, some of the most common GO biological processes were related to cation transport, potassium ion transport, ion transmembrane transport and nervous system development. Further enrichment and ontology analysis to these biological processes of the wound healing will be given in the sequel.

### Significant pathways in the wound healing process

The presence of temporally co-regulated proteins may imply the involvement of common biological pathways in response to TBI. Therefore, to evaluate the biological functions of temporally co-regulated proteins, pathway analysis was conducted using PANTHER. Upon the results, the roles of these significantly enriched pathways of groups (A, N, and P) in the zebrafish TBI model are investigated.

#### Significantly enriched pathways of group A: the acute inflammation and immune response in the wound healing process

Examples of the pathways are given in Fig. S4 in File S1 which are also primarily involved in the acute inflammation and immune response to TBI (The detail is enlisted in Table S2 in File S1).

1. Endogenous cannabinoid signaling: An endogenous cannabinoid (2-AG) has been implicated to affect the neuroprotective mechanism immediately after TBI [26]. The endo-cannabinoid system is also involved in pain relief, the blocking of working memory, immune responses, etc. The endo-cannabinoid system has therefore been suggested as a potential target for acute therapies for TBI [27].
2. PI3K/PKB pathway: Phosphoinositide 3-kinase (PI3K) catalyzes the production of phosphatidylinositol-3,4,5-trisphosphate, which is involved in several crucial biological processes, including cell survival, regulation of gene expression and cell metabolism, and cytoskeletal rearrangements. A study has reported that PKB, which belongs to group A, modulates the macrophage inflammatory response to *Francisella* infection and confers a survival advantage in mice [28].
3. Heterotrimeric G-protein signaling pathway: As signal transducers, G proteins communicate signals from many hormones, neurotransmitters, and other signaling factors [29] occurring ubiquitously in the cerebellar wound healing process.

#### Significantly enriched pathways of group P: positive correlation with ZMI in the wound healing process

The primarily involving pathways are given in Fig. S5 in File S1 and investigated in the following (The detail is enlisted in the Table S3 in File S1). They either played important roles in cytoskeleton regulation, angiogenesis and inflammation, or reflected the cellular processes by which behavioral disability increased initially and then decreased.

1. Cell cycle and DNA replication: An ordered series of events led to the replication of cells and a series of integrated protein-protein and protein-DNA interactions and enzymatic reactions, to ensure the accuracy of DNA replication. That may implicate the occurrence of angiogenesis and neurogenesis in the damaged site.
2. Parkinson's disease pathway, Huntington's disease pathway, and Alzheimer's disease-presenilin pathway: Parkinson's, Huntington's, and Alzheimer's diseases are three of the most notorious neurodegenerative diseases. Patients with neurodegenerative diseases suffer from progressive loss of structure or function of neurons, including the death of neurons. Research is currently identifying many similarities at a sub-cellular level between these diseases. For example, mitochondrial dysfunction and oxidative stress play a causal role in neurodegenerative disease pathogenesis [30]. The appearance of these pathways reveals the relationship between the secondary damage of cerebellar injury and neurodegenerative diseases.
3. Inflammation mediated by chemokine and cytokine signaling pathway, T cell activation, and B cell activation: Upon binding to a family of G-protein coupled with seven-transmembrane receptors, chemokines control and manage trafficking and migration of immune cells. This pathway illustrates chemokine-induced adhesion and migration of leukocytes, resulting in infiltration to the tissue and transcriptional activation to enable the recruitment of more leukocytes for protection from infection in the cerebellar wound healing process [31], [32]. Inhibiting specific chemokines and receptors could therefore prevent excessive recruitment of leukocytes to effectively control the scale of inflammation. T and B cells are the most abundant lymphocytes. T cell activation refers to a process in which mature T cells, which express antigen-specific T cell receptors on their surfaces, recognize their cognate antigens and respond by entering the cell cycle, secreting cytokines or lytic enzymes, and initiating the cell-based functions of the immune system. B cell activation entails the process by which a pre-B cell matures into a plasma cell. The signaling pathways for this inflammation resolution in the wound healing process are complex.
4. Cytoskeletal regulation by Rho GTPase and Axon guidance: In cerebellum regeneration processes such as wound healing and angiogenesis, many cell types, including neural stem cells, leukocytes, lymphocytes, fibroblasts, neuronal cells, epithelial cells and endothelial cells, need to change cell morphology for migration from stem cell niche and extravasation from the vascular in the wound healing process. Changing in cell morphology is a multistep process involving changes in the cytoskeleton, cell-substrate adhesions and extracellular matrix [33]. The “cytoskeletal regulation by Rho GTPase” pathway is such a change, regulated by Rho GTPase. The integrin signaling pathway is triggered when integrins in the cell membrane bind to extracellular matrix components [34]. Furthermore, axons are guided along specific tracts by attractive and repulsive cues during the establishment of the nervous system network in the cerebellar wound healing process.

#### Significantly enriched pathways of group N: negative correlation with ZMI in the wound healing process

Below are the pathways that were significantly enriched by the genes in group N (Fig. S6 and Table S4 in File S1). These neurotransmitter-related pathways appeared to be inhibited during the wound healing process. They may not be as crucial for regenerability as the above mentioned pathways, but they support the functional recovery of neural transmission in cerebellar wound healing process.

Some examples of these neurotransmitter-related pathways are: Beta1 adrenergic receptor signaling pathway, Beta2 adrenergic receptor signaling pathway, Synaptic vesicle trafficking, Muscarinic acetylcholine receptor 2 and 4 signaling pathway, Metabotropic glutamate receptor group II pathway, 5-HT1 type receptor mediated signaling pathway, Metabotropic glutamate receptor group III pathway, Enkephalin release pathway, GABA-B receptor II signaling pathway, Beta3 adrenergic receptor signaling pathway, Dopamine receptor mediated signaling pathway, Opioid prodynorphin pathway, Opioid proenkephalin pathway, 5-HT4 type receptor mediated signaling pathway, Muscarinic acetylcholine receptor 1 and 3 signaling pathway, Ionotropic glutamate receptor pathway, and Opioid proopiomelanocortin pathway. These pathways are all related to neurotransmitters and receptors in neural transmission and their roles in restoration and neurogenesis of the cerebellar wound healing process need further elaboration.

### Cross-talks between the three groups of proteins of PPI network in the cerebellar wound healing process

Moreover, the orchestrated interactions (cross-talks) among pathways are more important than themselves. The cross-talks among signal/regulatory pathways have been discussed to coordinate for efficient inflammatory responses for different stimuli in infectious process [35], [36]. Therefore, at first, we examine the cross-talks between the three groups of proteins, extracted from our dynamic PPI network (in Fig. 5), in the cerebellar wound healing process.

**Figure 7:**
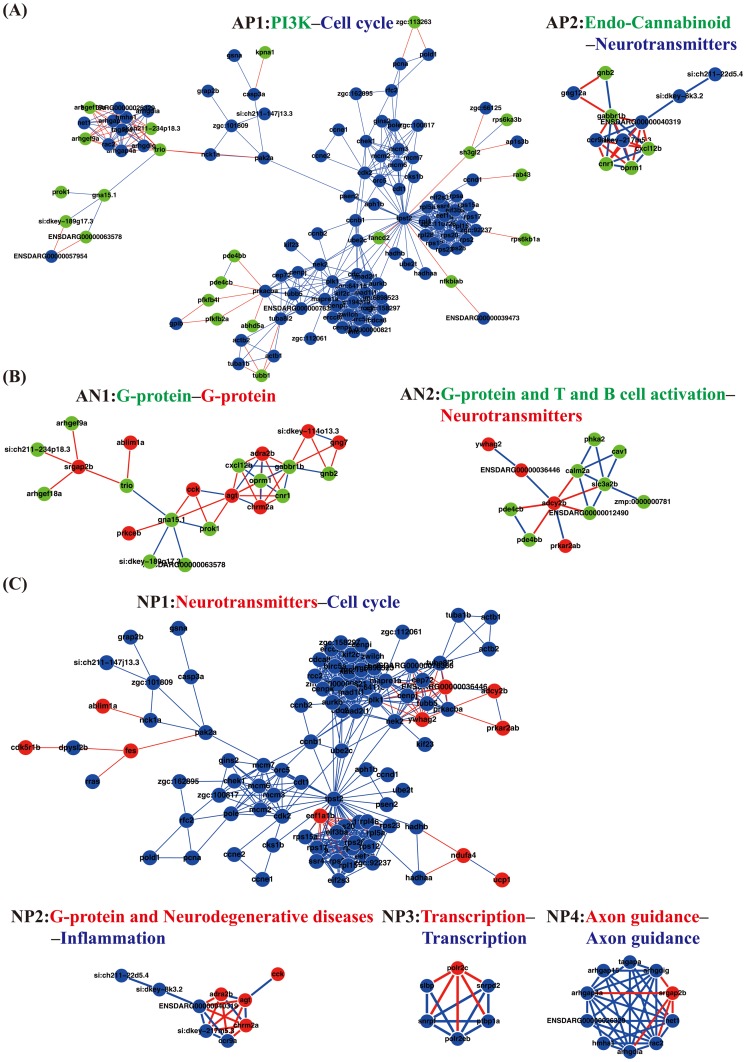
Cross-talks among significantly enriched pathways in the three significantly temporal groups of proteins. (A) Cross-talks between groups A and P: Cross-talks between PI3K pathways (enriched in group A) and the cell cycle (enriched in group P) dominated sub-network AP1. Cross-talks between endo-cannabinoid pathways (enriched in group A) and neurotransmitter-related pathways (enriched in group P) dominated sub-network AP2. (B) Cross-talks between groups A and N: Cross-talks between G-protein pathways (enriched in proteins from both groups A and N) dominated sub-network AN1. The cross-talks between G-proteins and T and B cell activation pathways (enriched in group A) and neurotransmitter-related pathways (enriched in group N) dominated the sub-network AN2. (C) Cross-talks between groups N and P: Cross-talks between neurotransmitter-related pathways (enriched in group N) and cell cycle pathways (enriched in group P) dominated sub-network NP1. Cross-talks between G-protein and neurodegenerative diseases pathways (enriched in group N) and inflammation pathways (enriched in group P) dominated sub-network NP2. Cross-talks between transcription pathways (enriched in both groups N and P) dominated sub-network NP3. Cross-talks between axon guidance pathways (enriched in both groups N and P) dominated sub-network NP4. Red lines indicate the cross-talks between groups and are listed in Table S5 in File S1.

In groups A and P, i.e., the proteins whose temporary expression profiles are positively correlated with the ZMI and the proteins in the acute phase, there are two major sub-networks (AP1 and AP2) emerging from our PPI network (see Fig. 7A; ZFIN symbols for the edges between two groups are given in Table S5 in File S1). In the sub-network AP1, the cross-talks between the PI3K pathway enriched in group A and cell cycle pathway and immune-related pathway enriched in group P are observed. This observation supports our understanding that PI3K pathways are involved in several crucial recovery processes, including cell proliferation and immune response in cerebellar wound healing process. In the sub-network AP2, the cannabinoid pathway cross-talks with neurotransmitter-related pathways. The cannabinoid pathway has shown its importance in neurogenesis [26], [37]. This may imply that neurotransmitter-related pathways should play a role in neurogenesis, which may be regulated by cannabinoid pathway and has not been fully explored [38], [39].

In groups A and N, there were also two major sub-networks (AN1 and AN2) (Fig. 7B; ZFIN symbols for the edges between two groups are given in Table S5 in File S1). The sub-network, AN1, contains interactions among cellular communication pathways because G-protein pathways are enriched in both groups. In the sub-network AN2, G-protein pathways and T and B cell activation pathways enriched in group A have links to neurotransmitter-related pathways enriched in group N. This cross-talk between immune system and neurotransmitters is an interesting area in cerebellar wound healing process [40]–[42].

**Figure 8:**
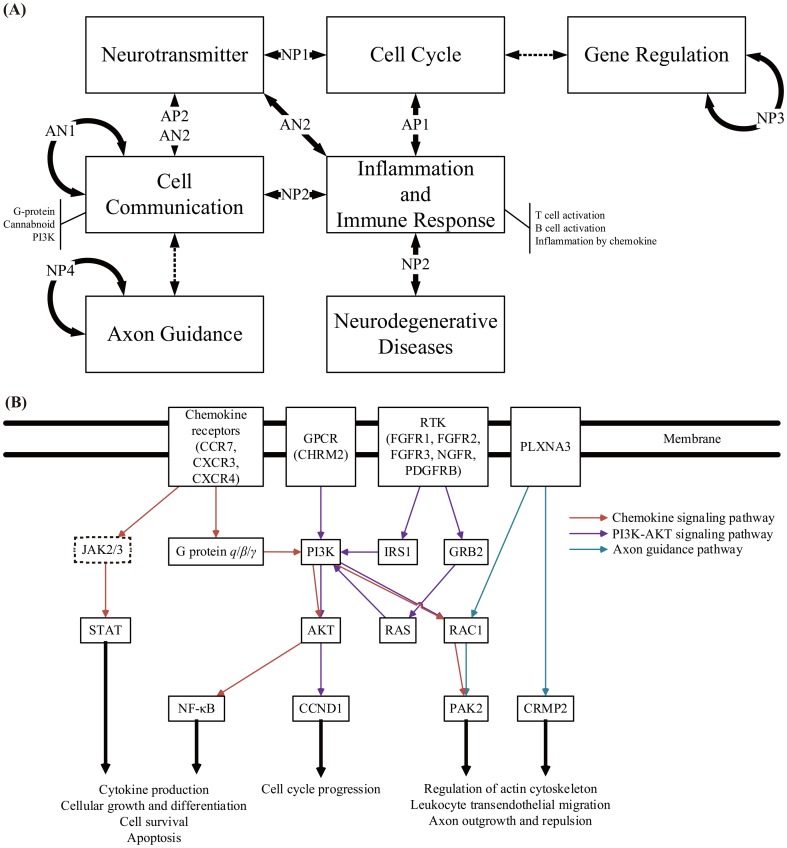
Schematic diagram of wound healing-related cellular pathways and their cross-talks among inflammation, neurogenesis, and angiogenesis. (A) Pathways shown in blocks were enriched in the sub-networks (Fig. 7). Their cross-talks were observed in the cerebellar wound healing PPI network of zebrafish (solid arrows indicate cross-talks involving proteins in groups A, N, and P; dashed arrows indicate cross-talks involving proteins not in these groups). To establish cell communication and defense mechanisms (inflammation and immune response) after brain injury, cell communication was related to neurotransmitter-related pathways (through AP2 and AN2), the axon guidance pathway (through NP4 indirectly), and itself (through AN1). Defense mechanisms were related to the cell cycle pathway (through AP1), neurotransmitter-related pathways (through AN2), the cell communication pathway (through NP2), and neurodegenerative diseases (through NP2). Cell cycle pathways also interacted with gene regulation pathways through NP3 indirectly. (B) The cross-talks are found among pathways of inflammation, neurogenesis, and angiogenesis in cerebellar wound healing process. Inflammation is mediated by chemokine signaling pathway. New neural and vascular cells generation is mediated by PI3K-AKT signaling pathway and axon guidance pathway. PI3K in chemokine signaling pathway also regulates the PI3K-AKT signaling pathway and axon guidance pathway. PAK2 in axon guidance pathway also maintains the stability of vascular.

Third, there are four major sub-networks in groups N and P (NP1 to NP4) (Fig. 7C; ZFIN symbols for the edges between two groups are given in Table S5 in File S1). In the sub-network NP1, neurotransmitter-related pathways have links to the cell cycle pathway, which constitutes another interesting cross-talks [38], [39]. In the sub-network NP2, inflammation-related pathways of group N have cross-talks with the G-protein and Alzheimer's pathway. Neurodegenerative diseases are often associated with prolonged brain injury. However, the molecular mechanisms by which the prolonged brain injury could induce neurodegenerative diseases are still unclear. Based on our network, it suggests that inflammation may be involved in the origination of neurodegenerative diseases. The sub-network NP3 contains many gene regulatory interactions, e.g., transcriptions, are enriched in both groups. By examining adjacent nodes of NP3, we find that the sub-network NP3 links to the cell cycle pathway through nodes that were not included in the three groups. NP3 may play a role in regulating and communicating other genes that were not belong to the three groups. The sub-network NP4 is dominated by axon guidance pathway, which can be seen as an indication of neurogenesis or functional recovery in cerebellar wound healing process. By examining adjacent nodes of NP4, axon guidance pathway indirectly links to the G-protein pathways through nodes not including in the three groups. In summary, we develop a schematic diagram of cross-talks among the pathways enriched in the three group that may explain the underlying mechanism of the cerebellar wound healing process (Fig. 8A).

Generally, the cross-talks exist not only in these three groups but also in the whole PPI network. Next, by examining the cross-talks of the following three pathways (Fig. 8B) significantly enriched in the whole network, we find connection among inflammation, neurogenesis, and angiogenesis, which are essential for the restoration from the injured brain. In the chemokine signaling pathway, a pro-inflammatory pathway, chemokine (C-X-C motif) receptor 3 and 4 (CXCR3 and CXCR4) and chemokine (C-C motif) receptor 7 (CCR7) are found in our network and viewed as a start point to activate a series of inflammatory events (e.g., activations of STAT and NF-κB signaling pathways). The PI3K is in the middle of the pathway and also involved in another pathway, PI3K signaling pathway. In the PI3K signaling pathway, several receptor tyrosine kinases (FGFR, NGFR, and PDGFR), insulin receptor substrate 1 (IRS1), cholinergic receptor muscarinic 2 (CHRM2), and growth factor receptor-bound protein 2 (GRB2) are all in our network. The information will be transduced to AKT through PI3K, and then promote cell cycle progression through cyclin D1 (CCND1), which stands for a sign for new cell generation. More interestingly, the PI3K is also functional in axon guidance pathway through regulating cytoskeleton. In the axon guidance pathway, in particular, the semaphorins-related ones, integrin beta 1 (ITGB1) and plexin A3 (PLXNA3) in our network are receptors for semaphorins and activate downstream regulators: ras-related C3 botulinum toxin substrate 1 (RAC1), dihydropyrimidinase-like 2 (DPYSL2 or CRMP2), and p21 protein (Cdc42/Rac)-activated kinase 2 (PAK2). PAK2 is involved in the vascular stabilization, which may be an important function after angiogenesis. DPYSL2 controls branching guidance and number of nurites. PLXNA3 is related to the morphogenesis of motor neurons and thus could have contributions to the recovery of zebrafish movement. Through this pathway validation, it confirms our network and reveal the relationships among inflammation, neurogenesis, and angiogenesis in cerebellar wound healing process from the aspect of pathways cross-talks.

## Discussion

TBI is one of the leading causes of disability in the USA [1]. However, the complex and intricate nature of the brain makes it very difficult to address this problem. To date, there are no FDA-approved pharmacological therapies [43]. Nevertheless, several recent advances have been made, through mass spectrometry, bioinformatics tools and systems biology, in discovering biomarkers which could serve as potential therapy targets [44].

In this study, we used zebrafish, which can recover from cerebellum injuries, to investigate potential signaling pathways and their cross-talks that may be crucial for TBI recovery and can also constitute useful biomarkers. Based on a set of time-course microarray experiments and the integration of omic data extracted from databases, a zebrafish PPI network of cerebellum wound healing processes was built. By analyzing the temporal expression patterns, three major protein groups (the acute, positively and negative related groups, i.e. Groups A, P, and N) are identified. Within each group, several significantly enriched pathways are found, and their relationships with the cerebellar wound healing process are confirmed through literature survey. The cross-talks of these significantly enriched pathways are revealed by using the constructed zebrafish PPI network of cerebellum wound healing process (see Fig.8A).

After injury of the cerebellum, cellular communication (i.e. G-protein and endo-cannabinoid pathways) and defense mechanisms (inflammation and immune responses) are provoked to coordinate the subsequent recovery processes. G-protein pathways and endo-cannabinoid pathways interact with neurotransmitter-related pathways (through sub-networks AP2 and AN2), after which those neurotransmitter-related pathways cause the activation of cell cycle pathways (through sub-network NP1), which is crucial in neurogenesis. Meanwhile, the G-protein pathways also indirectly initiate axon guidance which is maintained by cross-talks between groups N and P (sub-network NP4). The defense mechanisms, i.e. inflammation and immune responses, interact with cell cycle pathways and neurotransmitter-related pathways for restoration through sub-networks AP1 and AN2, respectively. Inflammation is interpreted as a necessary and sufficient condition for neurogenesis [45]. Hence, there should be cross-talks between the inflammation and cell cycle pathways for coordination in cerebellar wound healing process. Moreover, inflammation also seems to be connected with neurodegenerative diseases, e.g. Alzheimer's, Parkinson's and Huntington's diseases (through sub-network NP2), which are common in brain injury patients [46]. The cross-talks between immune responses and neurotransmitter-related pathways, which have also been reported in the literature, may provide an opportunity for TBI therapy. The following pathway validation reconfirms our PPI network and reveals the cross-talks among inflammation neurogenesis, and angiogenesis, which are crucial in the TBI recovery. The PI3K (Fig. 8B) adapts the information from chemokines, growth factors, and G-protein coupled receptors and further transduces the information to NF-κB, cyclins, and PAK2. It seemly plays a central role to regulate cytokine production, cell cycle progression, and axon outgrowth. Besides PI3K, we also found some plausible targets for further experimental designs, for example, the steroid biosynthesis, the hormone biosynthesis, and the notch signaling pathway, which are all enriched in our network.

The systems biology methodology can systematically analyze high-throughput data, and has also been suggested as a promising way to discover biomarkers for TBI [47]. Our refined PPI network reveals the cross-talks between several pathways, including some well-known cross-talks to coordinate between the immune response and cell communication processes for regeneration. Our results also indicates cross-talks between the immune response process and neurodegenerative diseases, which have been demonstrated by Feala et al. [47]. Neurotransmitters may also be involved in the TBI recovery process through their effect on cell cycle regulations [38], immune responses [48], and cell communication [49]. The roles of the neurotransmitter-related pathways in the TBI process have been less addressed. It is therefore interesting to study the roles of neurotransmitters in neuron regeneration. Finally, the cross-talks and plausible nodes identified in this study will be helpful in deciphering the molecular interactions between these pathways or cells in the wound healing process, and make human CNS regeneration possible in the future.

## Supporting Information

File S1 **Combined supporting information file containing the following**: Method S1. Dynamic model of the wound healing-related cellular PPI network. Method S2. Interaction parameter identification using the time series microarray data. Method S3. Determination of significant interaction pairs. Table S1. RNA QA/QC information. Table S2. The significantly enriched pathways in group A. Table S3. The significantly enriched pathways in group P. Table S4. The significantly enriched pathways in group N. Table S5. ZFIN symbols of nodes in the sub-networks. Figure S1. The wrapped needle and the sagittal brain sections after injury. Figure S2. 3D image of blood vessels and proliferating cells during regeneration. Figure S3A. The published time course microarray data for validation. Figure S3B. The published time course microarray data for validation. Figure S4. Examples of the over-represented pathways enriched by proteins from group A and involved in the acute inflammation and immune response during the wound healing process. Figure S5. Examples of the over-represented pathways enriched by proteins from group P and positively correlated with ZMI. Figure S6. Examples of the over-represented pathways enriched by proteins from group N and negatively correlated with ZMI.
(DOCX)

File S2 **The list of protein-protein interactions for** **Fig.5** **.**
(XLSX)
