# Supplementary material for: On the Crucial Cerebellar Wound Healing-Related Pathways and Their Cross-Talks after Traumatic Brain Injury in Danio rerio
Source: PLoS One. 2014 Jun 13;9(6):e97902. doi: 10.1371/journal.pone.0097902 (PMC4057083; doi:10.1371/journal.pone.0097902)
Supplement: File S1 — Combined supporting information file containing the following: Method S1. Dynamic model of the wound healing-related cellular PPI network. Method S2. Interaction parameter identification using the time series microarray data. Method S3. Determination of significant interaction pairs. Table S1. RNA QA/QC information. Table S2. The significantly enriched pathways in group A. Table S3. The significantly enriched pathways in group P. Table S4. The significantly enriched pathways in group N. Table S5. ZFIN symbols of nodes in the sub-networks. Figure S1. The wrapped needle and the sagittal brain sections after injury. Figure S2. 3D image of blood vessels and proliferating cells during regeneration. Figure S3A. The published time course microarray data for validation. Figure S3B. The published time course microarray data for validation. Figure S4. Examples of the over-represented pathways enriched by proteins from group A and involved in the acute inflammation and immune response during the wound healing process. Figure S5. Examples of the over-represented pathways enriched by proteins from group P and positively correlated with ZMI. Figure S6. Examples of the over-represented pathways enriched by proteins from group N and negatively correlated with ZMI. (DOCX) [file pone.0097902.s001.docx]

**Supplementary material**

**Method S1. Dynamic model of the wound healing-related cellular PPI network**

In the above candidate PPI network, the candidate interactions could be depicted as a dynamic system, in which the interactive proteins and mRNA are the inputs and the protein activities are the outputs. In particular, given a target protein *p* that interacts with *N* proteins in the candidate PPI network, the dynamic model of the protein *p* is described as follows:

$$y_{p}\left[t+1\right]=y_{p}\left[t\right]+\sum_{q=1}^{N} b_{pq}y_{p}\left[t\right]y_{q}\left[t\right]+\alpha_{p}x_{p}\left[t\right]-\beta_{p}y_{p}\left[t\right]+\omega_{p}\left[t+1\right]$$

where *y_p_*[*t*] and *y_q_*[*t*] represent the protein activity level of the target protein *p* and the *q*-th protein interacting with *p* at time *t*, respectively; *b_pq_* denotes the ability of the *q*-th interactive protein to interact with *p*; *α_p_* denotes the translation effect from mRNA to *p*; *x_p_*[*t*] represents the mRNA expression level of *p*; *β_p_* indicates the degradation effect of *p*; and *ω_p_*[*t*+1] is stochastic noise. The rate of PPI is proportional to the product of the concentrations of the two proteins involved ([Alon, 2007](#_ENREF_2)), i.e. it is proportional to the probability of molecular collisions between two proteins. The interaction is therefore modeled as a nonlinear multiplication scheme. For example, in the PPI network, the phosphorylation of *y_p_*[*t*] by kinase *y_q_*[*t*] is proportional to the product of the concentration of kinase *y_q_*[*t*] and its substrate *y_p_*[*t*] ([Alon, 2007](#_ENREF_2)). The biological meaning of equation (1) is that the activity level of *p* at time *t*+1 is attributed to its activity level at time *t*, plus the effects of interactions with *N* interactive proteins, plus the translation product from the mRNA, minus the degradation effect, plus any stochastic noise. Because of the undirected nature of protein interactions, there is no direction between interacting proteins in the candidate PPI network. The interaction parameter *b_pq_*, translation parameter *α_p_* and decay rate *β_p_* were estimated from the microarray data, as described below.

**Method S2. Interaction parameter identification using the time series microarray data**

The interaction parameters for the dynamic network model were identified using the microarray data collected as described above. The interaction parameters were identified using a least squares parameter estimation formula ([Coleman & Hulbert, 1989](#_ENREF_3)). Equation (1) can be rewritten as follows:

where *ϕ_p_*[*t*] indicates the regression vector, which can be computed directly from microarray data points, and *θ_p_* is the parameter vector to be estimated. The dataset used for parameter estimation was small, so to avoid overfitting, the cubic spline method ([De Boor, 2001](#_ENREF_4); [Jaakkola, 2002](#_ENREF_5)) was used to interpolate extra time points for the gene expression data. For simplicity, at different time points *t*= 1, …, *L*, equation (2) can be presented as follows:

$$Y_{p}=\Phi_{p}\theta_{p}+\Omega_{p}$$

where *Y_p_*=[*y_p_*[2] *y_p_*[1] ⋯ *y_p_*[*L*]]*^T^*, Φ*_p_*=[*ϕ_p_*[1] *ϕ_p_*[2] ⋯ *ϕ_p_*[*L*–1]], and *L* is the number of data points of microarray data after cubic spline interpolation. The least squares parameter estimation routine can then be formulated as follows:

$\mathop{\text{min}}\limits_{\theta_{p}} \left\|Y_{p}-\Phi_{p}\theta_{p}\right\|_{2}^{2}$.

Because there was no large-scale measurement of protein activities available, mRNA expression profiles in Fig. 2 were used to substitute for protein activity levels when identifying the interaction parameters. Although mRNA expression levels do not reflect protein expression levels exactly, there are partially positive correlations between them ([Newman et al, 2006](#_ENREF_8); [Orntoft et al, 2002](#_ENREF_9)). The least squares minimization formula (4) can be solved using the active set method for quadratic programming ([Coleman & Hulbert, 1989](#_ENREF_3)). The PPI parameter *b_pq_* was estimated for each protein in the candidate PPI network using the time series microarray data, i.e. through the estimated interaction abilities. However, since the candidate PPI network was constructed using data from a variety of biological experiments under a range of conditions, or inferred from orthology data, it contained many PPIs irrelevant to the wound healing process. The estimated interaction parameters *b̂_pq_* were therefore pruned using the model order (true interaction number) detection method, as described below.

**Method S3. Determination of significant interaction pairs**

When the PPI parameters *b̂_pq_* were identified, Akaike’s Information Criterion (AIC) ([Akaike, 1974](#_ENREF_1); [Johansson, 1993](#_ENREF_6)) was used to select model order and also identify significant interactions in the cerebellar wound healing-related PPI network, i.e., to determine the number *N* for target protein *p* in equation (1). AIC decreases as the residual error decreases and increases with the number of interactions (model complexity). As the expected residual error decreases with increasing interaction numbers for non-adequate model complexities, AIC should reach a minimum around the correct PPI number ([Akaike, 1974](#_ENREF_1); [Johansson, 1993](#_ENREF_6)). Therefore, AIC can be used to detect model order (number of PPIs) based on the protein interaction abilities *b̂_pq_* identified above. In this way we used AIC model order detection to prune the candidate PPI network one protein by on protein to obtain the refined PPI network using the time series microarray data and achieve a realistic PPI network.

**Table S1. RNA QA/QC information**

| Sample Name | RNA conc. (ng/μL) | RNA quantity (ng) | OD260/280 Ratio | Bioanalyzer chip lane location | 28S/18S Ration | RIN |
| --- | --- | --- | --- | --- | --- | --- |
| 6 hpl | 227.12 | 2044.08 | 2.12 | 1 | 1.8 | 9.4 |
| 1 dpl | 279.09 | 2511.81 | 2.11 | 2 | 1.3 | N/A |
| 3 dpl | 429.08 | 3861.72 | 2.12 | 3 | 1.0 | 9.4 |
| 6 dpl | 308.67 | 2778.03 | 2.13 | 4 | 1.3 | 9.7 |
| 10 dpl | 372.76 | 3354.84 | 2.14 | 5 | 1.4 | 9.6 |
| 15 dpl | 510.94 | 4598.46 | 2.13 | 6 | 0.9 | 9.1 |
| 21 dpl | 354.08 | 3186.72 | 2.1 | 7 | 1.5 | 8.6 |
| 28 dpl | 468.07 | 4212.63 | 2.13 | 8 | 1.5 | 9.3 |
| control | 576.71 | 5190.39 | 2.17 | 9 | 1.4 | 9.0 |

**Table S2. The significantly enriched pathways in group A.**

| Pathways | p-value |
| --- | --- |
| Endogenous_cannabinoid_signaling | 7.17E-06 |
| PI3 kinase pathway | 1.87E-05 |
| Androgen/estrogene/progesterone biosynthesis | 1.90E-05 |
| GABA-B_receptor_II_signaling | 3.47E-05 |
| Heterotrimeric G-protein signaling pathway-Gq alpha and Go alpha mediated pathway | 4.50E-05 |
| Thyrotropin-releasing hormone receptor signaling pathway | 2.88E-04 |
| Insulin/IGF pathway-mitogen activated protein kinase kinase/MAP kinase cascade | 6.87E-04 |
| Metabotropic glutamate receptor group II pathway | 1.26E-03 |
| TCA cycle | 1.69E-03 |
| Circadian clock system | 3.47E-03 |

**Table S3. The significantly enriched pathways in group P.**

| Pathways | p-value |
| --- | --- |
| Cell cycle | 4.94E-08 |
| Parkinson disease | 3.20E-05 |
| Integrin signaling pathway | 3.43E-05 |
| De novo pyrimidine deoxyribonucleotide biosynthesis | 2.68E-04 |
| Inflammation mediated by chemokine and cytokine signaling pathway | 4.15E-04 |
| Axon guidance mediated by semaphorins | 6.53E-04 |
| Cytoskeletal regulation by Rho GTPase | 8.56E-04 |
| T cell activation | 8.63E-04 |
| p53 pathway | 2.02E-03 |
| B cell activation | 3.29E-03 |
| Alzheimer disease-presenilin pathway | 5.94E-03 |
| Huntington disease | 5.98E-03 |

**Table S4. The significantly enriched pathways in group N.**

| Pathways | p-value |
| --- | --- |
| Muscarinic acetylcholine receptor 2 and 4 signaling pathway | 3.56E-10 |
| Heterotrimeric G-protein signaling pathway-Gi alpha and Gs alpha mediated pathway | 2.84E-08 |
| GABA-B_receptor_II_signaling | 9.11E-08 |
| Dopamine receptor mediated signaling pathway | 1.39E-07 |
| 5HT1 type receptor mediated signaling pathway | 2.63E-07 |
| Beta1 adrenergic receptor signaling pathway | 3.49E-07 |
| Beta2 adrenergic receptor signaling pathway | 3.74E-07 |
| Histamine H2 receptor mediated signaling pathway | 1.41E-06 |
| Endothelin signaling pathway | 4.66E-06 |
| Enkephalin release | 5.30E-06 |
| Metabotropic glutamate receptor group II pathway | 8.41E-06 |
| Muscarinic acetylcholine receptor 1 and 3 signaling pathway | 2.46E-05 |
| Beta3 adrenergic receptor signaling pathway | 5.54E-05 |
| Alpha adrenergic receptor signaling pathway | 6.49E-05 |
| Adrenaline and noradrenaline biosynthesis | 7.01E-05 |
| Metabotropic glutamate receptor group III pathway | 7.30E-05 |
| Opioid prodynorphin pathway | 1.07E-04 |
| 5HT4 type receptor mediated signaling pathway | 1.07E-04 |
| 5-Hydroxytryptamine biosynthesis | 1.24E-04 |
| Opioid proopiomelanocortin pathway | 1.29E-04 |
| Opioid proenkephalin pathway | 1.29E-04 |
| Synaptic_vesicle_trafficking | 1.54E-04 |
| Transcription regulation by bZIP transcription factor | 4.92E-04 |
| Heterotrimeric G-protein signaling pathway-Gq alpha and Go alpha mediated pathway | 5.89E-04 |
| Nicotine pharmacodynamics pathway | 7.76E-04 |
| Alzheimer disease-amyloid secretase pathway | 8.28E-04 |
| Oxytocin receptor mediated signaling pathway | 8.28E-04 |
| Thyrotropin-releasing hormone receptor signaling pathway | 9.08E-04 |
| Gonadotropin releasing hormone receptor pathway | 1.02E-03 |
| 5HT2 type receptor mediated signaling pathway | 1.15E-03 |
| Parkinson disease | 2.24E-03 |
| Hedgehog signaling pathway | 2.83E-03 |
| Apoptosis signaling pathway | 2.87E-03 |
| Histamine synthesis | 3.99E-03 |
| Inflammation mediated by chemokine and cytokine signaling pathway | 4.11E-03 |
| Cortocotropin releasing factor receptor signaling pathway | 4.41E-03 |

**Table S5. ZFIN symbols of nodes in the sub-networks**

| Sub-network | Group A | Group N |  | Group A | Group N |
| --- | --- | --- | --- | --- | --- |
| AN1 | cxcl12b | chrm2a |  | trio | srgap2b |
|  | prok1 | agt |  | gabbr1b | adra2b |
|  | gabbr1b | chrm2a |  | gabbr1b | si:dkey-114o13.3 |
|  | trio | ablim1a |  | oprm1 | chrm2a |
|  | gabbr1b | gng7 |  | arhgef18a | srgap2b |
|  | cnr1 | chrm2a |  | gna15.1 | prkceb |
|  | gna15.1 | cck |  | gnb2 | gng7 |
|  | si:ch211-234p18.3 | srgap2b |  | oprm1 | adra2b |
|  | cxcl12b | adra2b |  | cnr1 | adra2b |
|  | gnb2 | si:dkey-114o13.3 |  | gna15.1 | agt |
|  | cnr1 | agt |  | prok1 | cck |
|  | cxcl12b | agt |  | gabbr1b | agt |
|  | arhgef9a | srgap2b |  | oprm1 | agt |
|  |  |  |  |  |  |
| AN2 | pde4bb | adcy2b |  | calm2a | adcy2b |
|  | pde4cb | adcy2b |  | slc3a2b | adcy2b |
| Sub-network | **Group A** | **Group P** |  | **Group A** | **GroupP** |
| AP1 | trio | rac2 |  | si:ch211-234p18.3 | arhgap4a |
|  | sh3gl2 | tpst2 |  | arhgef9a | tagapa |
|  | trio | hmha1 |  | sh3gl2 | zgc:66125 |
|  | trio | arhgap15 |  | tubb1 | actb1 |
|  | pfkfb4l | prkacba |  | si:ch211-234p18.3 | arhgap15 |
|  | pde4bb | prkacba |  | nfkbiab | tpst2 |
|  | fancd2 | tpst2 |  | tubb1 | tuba8l2 |
|  | sh3gl2 | ap1s3b |  | pde4cb | prkacba |
|  | trio | nck1a |  | trio | pak2a |
|  | arhgef9a | arhgap4a |  | rab43 | ccnd1 |
|  | zgc:113263 | pcna |  | trio | tagapa |
|  | tubb1 | tuba1b |  | pfkfb2a | prkacba |
|  | si:ch211-234p18.3 | hmha1 |  | rps6kb1a | zgc:92237 |
|  | arhgef9a | hmha1 |  | pfkfb2a | gpib |
|  | si:ch211-234p18.3 | tagapa |  | si:ch211-234p18.3 | rac2 |
|  | arhgef9a | arhgap15 |  | zgc:113263 | pold1 |
|  | tubb1 | actb2 |  | arhgef18a | arhgap15 |
|  | arhgef18a | hmha1 |  | arhgef9a | rac2 |
|  | arhgef18a | rac2 |  | arhgef18a | arhgap4a |
|  | pfkfb4l | gpib |  | fancd2 | ube2t |
|  | arhgef18a | tagapa |  | kpna1 | casp3a |
|  | trio | arhgap4a |  |  |  |
|  |  |  |  |  |  |
| AP2 | cnr1 | ccr9a |  | cxcl12b | si:dkey-217m5.3 |
|  | gnb2 | gng12a |  | cnr1 | si:dkey-217m5.3 |
|  | gabbr1b | si:dkey-217m5.3 |  | gabbr1b | gng12a |
|  | oprm1 | si:dkey-217m5.3 |  | oprm1 | ccr9a |
|  | gabbr1b | ccr9a |  | cxcl12b | ccr9a |
| Sub-network | **Group N** | **Group P** |  | **Group N** | **GroupP** |
| NP1 | fes | rras |  | eef1a1b | zgc:92237 |
|  | prkar2ab | prkacba |  | eef1a1b | rpl5a |
|  | adcy2b | prkacba |  | eef1a1b | rps23 |
|  | fes | dpysl2b |  | eef1a1b | rps17 |
|  | cdk5r1b | dpysl2b |  | eef1a1b | rps28 |
|  | ndufa4 | hadhaa |  | eef1a1b | rps20 |
|  | ndufa4 | hadhb |  | eef1a1b | rps12 |
|  | eef1a1b | eef1g |  | eef1a1b | rpl4 |
|  | ywhag2 | cep72 |  | eef1a1b | rps2 |
|  | ablim1a | nck1a |  | ywhag2 | prkacba |
|  | fes | pak2a |  | ywhag2 | nek2 |
|  | eef1a1b | tpst2 |  | ywhag2 | cenpj |
|  | eef1a1b | rpl28 |  | ywhag2 | tuba8l2 |
|  | eef1a1b | rpl15 |  | ywhag2 | tubb5 |
|  | eef1a1b | rps15a |  | ywhag2 | mapre1a |
|  | eef1a1b | rpsa |  | ywhag2 | plk1 |
|  |  |  |  |  |  |
| NP2 | adra2b | ccr9a |  | chrm2a | si:dkey-217m5.3 |
|  | agt | ccr9a |  | adra2b | si:dkey-217m5.3 |
|  | agt | si:dkey-217m5.3 |  | chrm2a | ccr9a |
|  |  |  |  |  |  |
| NP3 | polr2c | polr2eb |  | polr2c | slbp |
|  | polr2c | snrpf |  | polr2c | snrpd2 |
|  | polr2c | ptbp1a |  |  |  |
|  |  |  |  |  |  |
| NP4 | srgap2b | arhgdia |  | srgap2b | rac2 |
|  | srgap2b | arhgap4a |  | srgap2b | net1 |
|  | srgap2b | arhgdig |  |  |  |


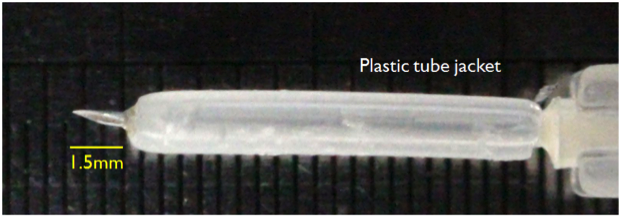

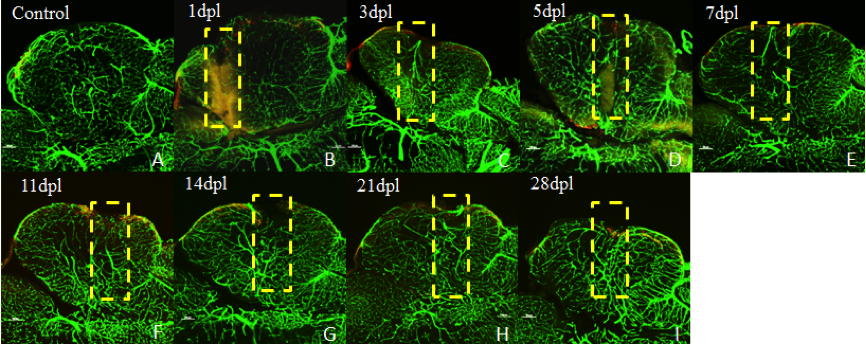


**Figure S1. The wrapped needle and the sagittal brain sections after injury.**

We controlled the injury depth by wrapping the needle with plastic tube and only left 1.5 mm needle tip for stabbing. Also the lesion depth with sagittal brain sections is examined. The white-dashed line surrounds the intact cerebellum, and the yellow-dashed rectangles in the other pictures point out the lesion depth. Lesion depth caused by modified needle only penetrates the cerebellum region, and does not wound other brain parts.


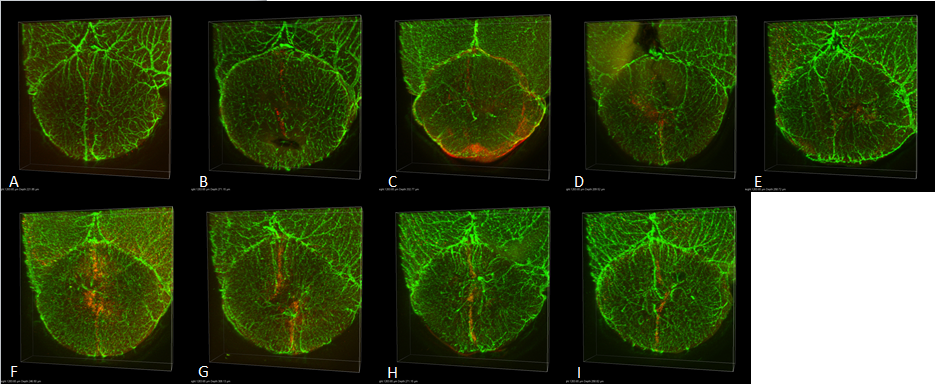


**Figure S2. 3D image of blood vessels and proliferating cells during regeneration.**

A: 3D image of blood vessels (green) and proliferating cells (red) before lesion. B-I: Represent 1-, 3-, 5-, 7-, 11-, 14-, 21- and 28- dpl cerebellum, respectively. B: After 1dpl, a wound can be clearly visible in cerebellum where the signals of blood vessels and proliferating cells are disappear. C-F: At 3- to 11- dpl, blood vessels are regrowth in the wound, and proliferating cells are accumulated to the wound. G: There is a gap in the wound where the blood vessels and proliferating cells are less. H-I: Blood vessels and proliferating cells fill in the wound and the PCNA signals are decrease if compare to 11- or 14- dpl.

Immunohistochemistry staining was performed following the standard procedure. Zebrafish brains were harvested and fixed with 4% paraformaldehyde (Merck) overnight at 4 °C. We then washed the zebrafish brains four times for 15 min in PBST. The brains were permeabilized in ice-cold acetone for 90 min at 4 C. They were then washed four times for 15 min with maleic acid buffer. Blocking buffer (2% BSA and 2% goat serum in maleic acid buffer) was used to block the brains for four hours at room temperature. Primary antibodies, rabbit anti-eGFP (1:600; Novus Biologicals, Littleton, CO, USA) and mouse anti-PCNA (sc-56, 1:50; Santa Cruz Biotechnology, Santa Cruz, CA, USA), were added to the blocking buffer incubate and stored overnight at 4°C. The samples were then washed four times for 30 min with maleic acid buffer. Secondary antibodies, goat anti-rabbit 488 (1:200; KPL, Gaithersburg, MD, USA) and goat anti-mouse 549 (1:200, KPL, Gaithersburg, MD, USA), were added to the blocking buffer to be incubated with the brains for 2 h. After that, brains were washed four times with maleic acid buffer for 30 min each time. Finally, brains were stored in FocusClear (CelExplorer Labs, Hsinchu, Taiwan) at 4 °C. Fluorescence images were taken using A1R confocal microscope (Nikon, Japan).


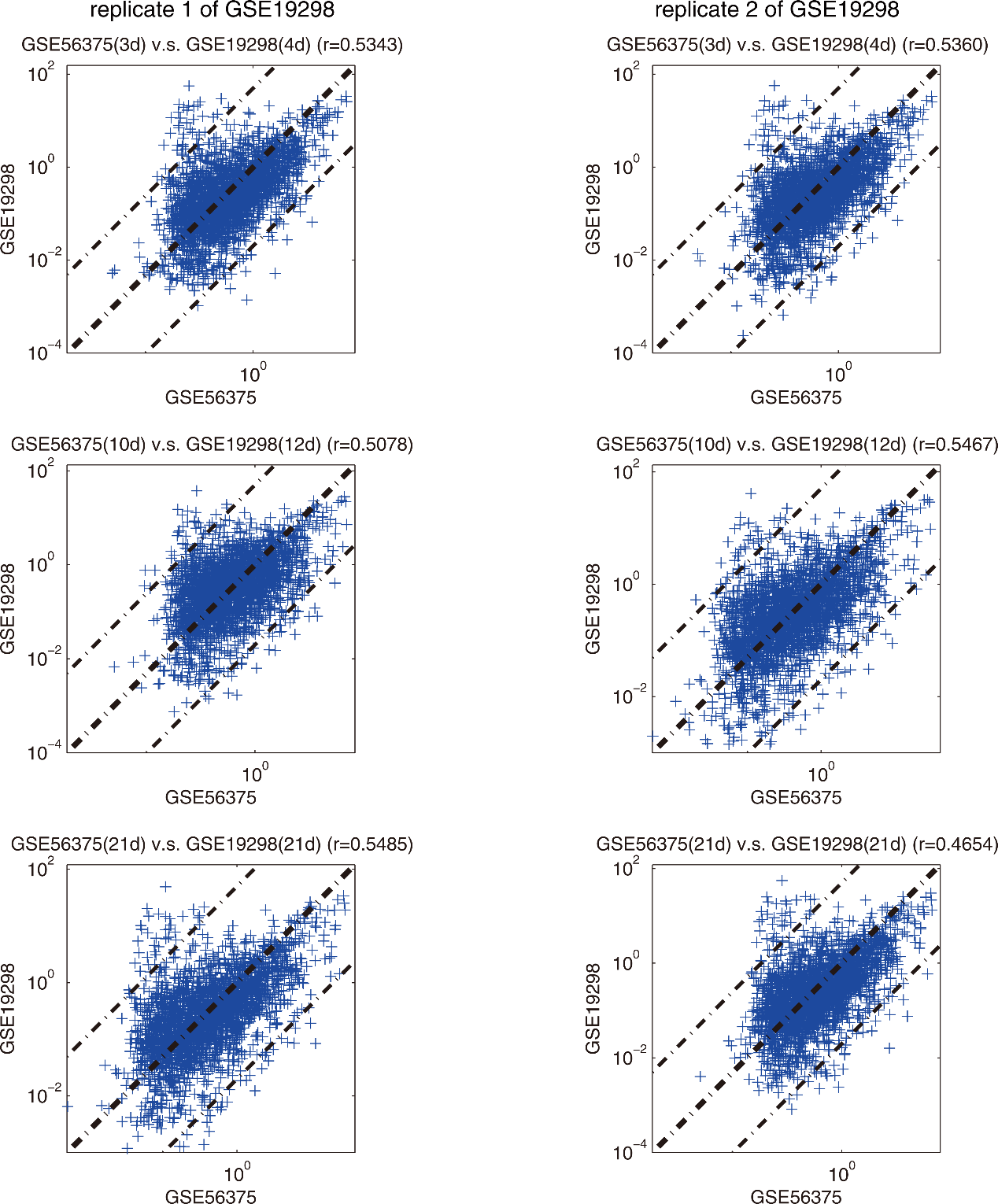


**Figure S3A. The published time course microarray data for validation.**

The published data of the optic nerve regeneration is GSE19298 ([McCurley & Callard, 2010](#_ENREF_7)). In this study, microarray analysis was performed on total RNA extracted from whole eye following optic nerve crush or sham surgery at defined intervals (4, 12, and 21 days). The x axis is our time course microarray data (after normalizing). The y axis is the published time course microarray data (after normalizing). The Pearson correlation coefficient is denoted as r.


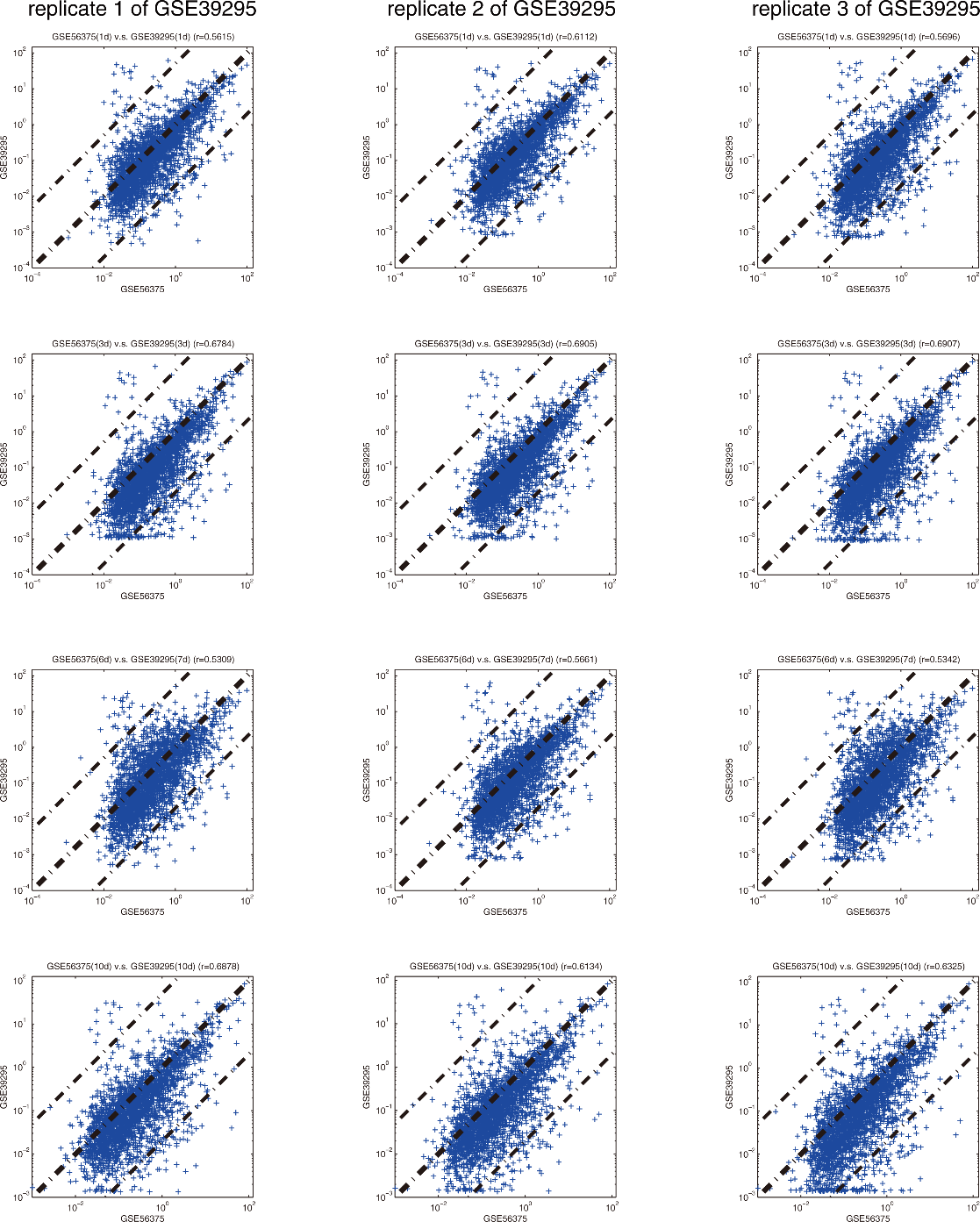


**Figure S3B. The published time course microarray data for validation.**

The published data of the spinal cord regeneration is GSE39295 (Hui et al, 2014). In this study, the spinal cord has been injured by crushing dorso-ventrally for 1 sec at the level of 15th/16th vertebrae. Later the wound were sealed by placing a suture. Both spinal cord injured and sham operated fish were allowed to regenerate and the progress of regeneration was observed after 1, 3, 7, 10 and 15 days of injury. Zebrafishes were anesthetized deeply for 5 minutes in 0.1% tricaine (MS222; Sigma, USA) and approximately 1 mm length of spinal cord both rostrally and caudally from injury epicenter were dissected out from 50-60 fishes in each batch and pooled for RNA extraction. The x axis is our time course microarray data (after normalizing). The y axis is the published time course microarray data (after normalizing). The Pearson correlation coefficient is denoted as r.


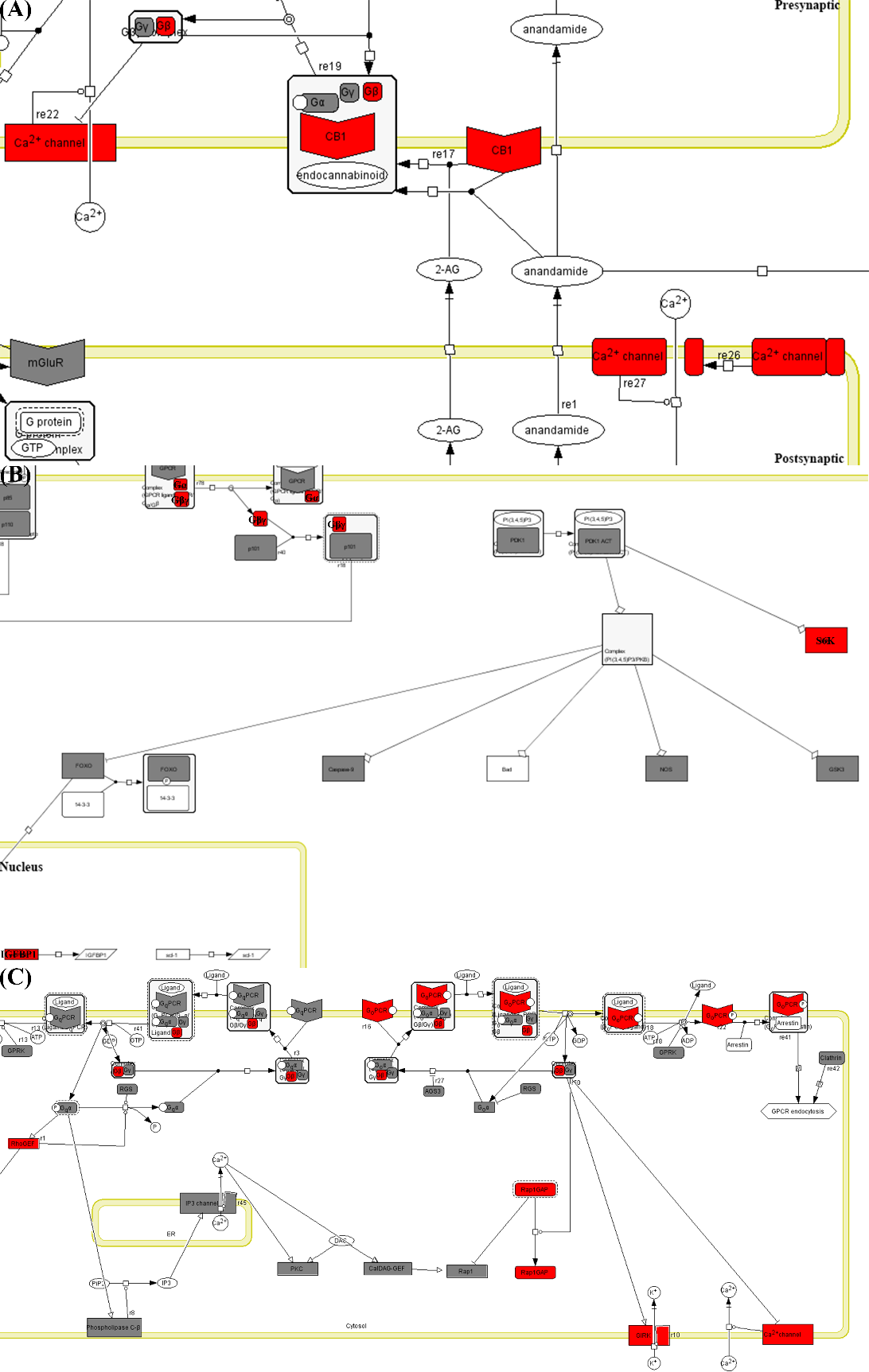


**Figure S4. Examples of the over-represented pathways enriched by proteins from group A and involved in the acute inflammation and immune response during the wound healing process.** These pathways were primarily involved in the acute inflammation and immune response to TBI. (A) Endogenous cannabinoid signaling pathway; (B) PI3 kinase pathway; (C) Heterotrimeric G-protein signaling pathway-Gq alpha and Go alpha mediated pathway. Proteins in group A are shown in red.


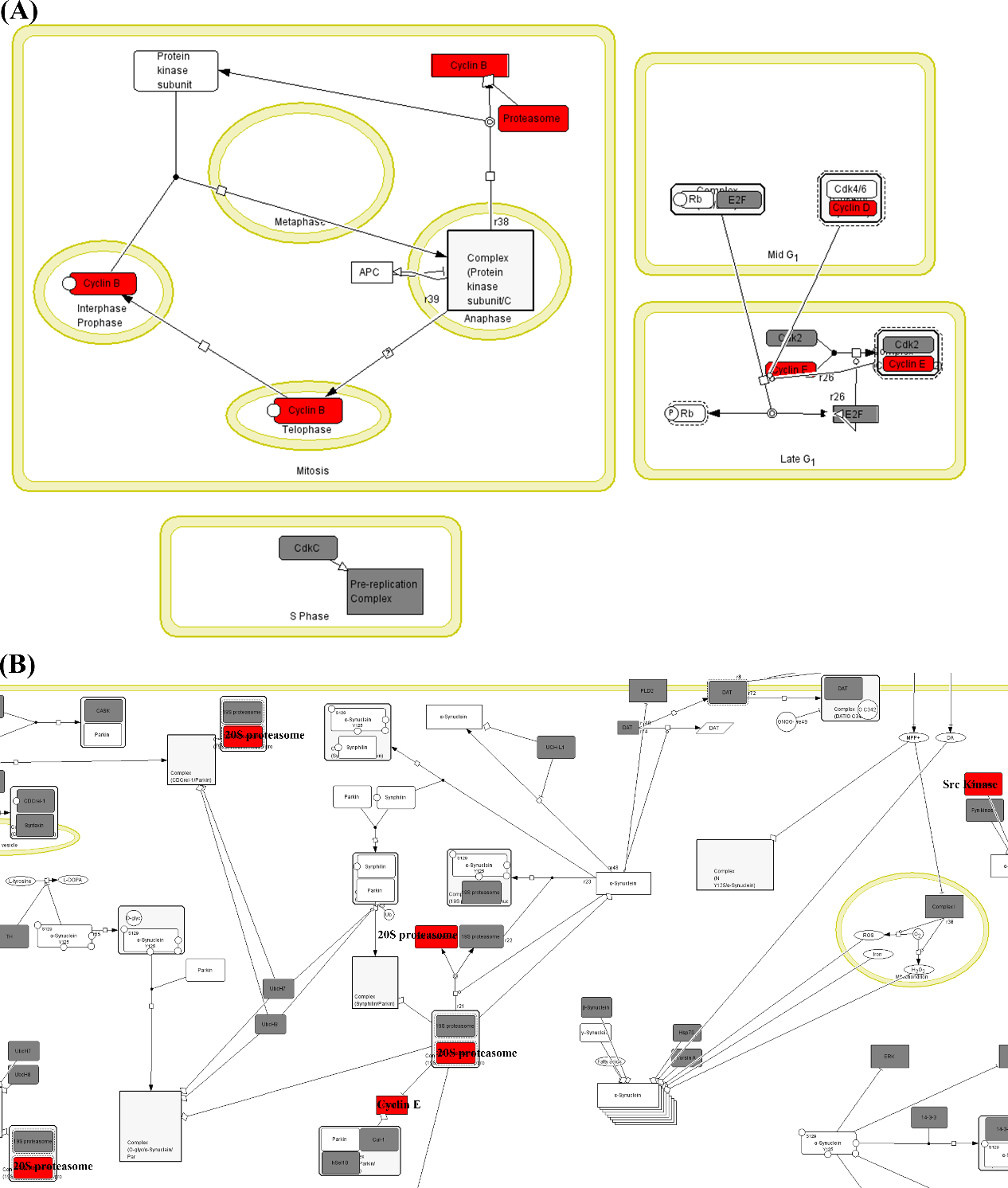


**Figure S5. Examples of the over-represented pathways enriched by proteins from group P and positively correlated with ZMI.** These pathways either played an important role in cytoskeleton regulation, angiogenesis and inflammation, or reflected the process in which behavioral disability increased initially and then decreased. (A) Cell cycle pathway; (B) Parkinson disease pathway. Proteins in group P are shown in red.


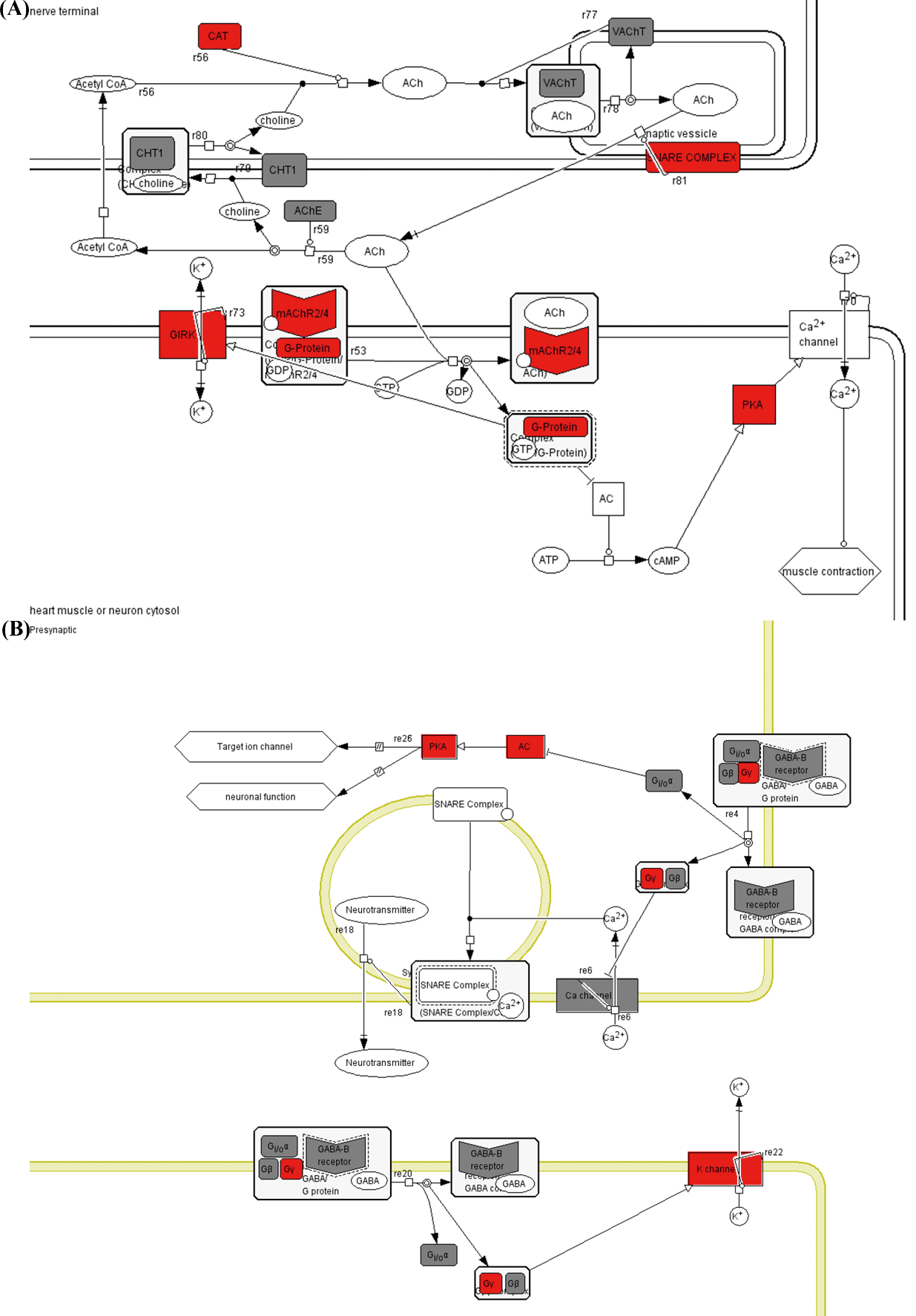


**Figure S6. Examples of the over-represented pathways enriched by proteins from group N and negatively correlated with ZMI.** These pathways support the functional recovery of neural transmission. (A) Muscarinic acetylcholine receptor 2 and 4 signaling pathway; (B) GABA-B receptor II signaling pathway. Proteins in group N are shown in red.

Referneces

Akaike H (1974) New Look at Statistical-Model Identification. *Ieee T Automat Contr* **Ac19:** 716-723

Alon U (2007) *An introduction to systems biology : design principles of biological circuits*, Boca Raton, FL: Chapman & Hall/CRC.

Coleman TF, Hulbert LA (1989) A Direct Active Set Algorithm for Large Sparse Quadratic Programs with Simple Bounds. *Math Program* **45:** 373-406

De Boor C (2001) *A practical guide to splines : with 32 figures*, Rev. edn. New York: Springer.

Jaakkola ZB-jaGGaDKGaTS (2002) A new approach to analyzing gene expression time series data. In *Proceedings of the sixth annual international conference on Computational biology*, pp 39-48.

Johansson R (1993) *System modeling and identification*, Englewood Cliffs, NJ: Prentice Hall.

McCurley AT, Callard GV (2010) Time Course Analysis of Gene Expression Patterns in Zebrafish Eye During Optic Nerve Regeneration. *Journal of experimental neuroscience* **2010:** 17-33

Newman JRS, Ghaemmaghami S, Ihmels J, Breslow DK, Noble M, DeRisi JL, Weissman JS (2006) Single-cell proteomic analysis of S-cerevisiae reveals the architecture of biological noise. *Nature* **441:** 840-846

Orntoft TF, Thykjaer T, Waldman FM, Wolf H, Celis JE (2002) Genome-wide study of gene copy numbers, transcripts, and protein levels in pairs of non-invasive and invasive human transitional cell carcinomas. *Mol Cell Proteomics* **1:** 37-45
